# “* … * [I]*f I can* [be] *infected now that means I am going to die … *”: an explorative study focusing on vulnerable, immunocompromised groups and caregivers experiences and perceptions of the Covid-19 pandemic in South Africa

**DOI:** 10.1080/17290376.2023.2187446

**Published:** 2023-03-21

**Authors:** Alicia North, Allanise Cloete, Shandir Ramlagan, Thabang Manyaapelo, Amukelani Ngobeni, Noloyiso Vondo, Derrick Sekgala

**Affiliations:** a South Australian Health and Medical Research Institute (SAHMRI), Registry of Older Australians (ROSA), South Australia; b HSC Division, HSRC, Pretoria, South Africa; c University of the North West, Potchefstroom, South Africa

**Keywords:** Covid-19, South Africa, vulnerable, immunocompromised, fear of infection, healthcare

## Abstract

In this paper, we explored how vulnerable, immunocompromised groups and caregivers of the elderly experienced and perceived the onset of the Covid-19 pandemic in South Africa. Semi-structured interviews were conducted remotely between the 5th andthe 18th of April 2020 in the three South African provinces hardest hit by Covid-19, namely Gauteng, KwaZulu-Natal and the Western Cape. In total, 60 qualitative key informant interviews and one focus group discussion were conducted. Study participants expressed concerns for elderly people and people with underlying health conditions because of their increased vulnerability to Severe Acute Respiratory Syndrome Coronavirus 2 (SARS-CoV-2). People living with HIV expressed an increased fear of infection following the advent of the Covid-19 pandemic in South Africa. The sidelining of healthcare services and stock-outs of medication proved to be an added concern in particular for vulnerable and immunocompromised groups. Overall, the data suggest that the fear of infection is ubiquitous for people who live in unstable environments such as overcrowded townships and informal settlements. Given the increased fears of infection brought on by the Covid-19 pandemic, the mental health of vulnerable communities and those caring for them becomes an added burden for people living in unstable environments.

## Introduction

In 2020, a new virus identified as the Severe Acute Respiratory Syndrome Coronavirus 2 (SARS-CoV-2), which was a new strain of virus in the coronavirus family, was identified (World Health Organization, 2020a). Globally people have built up immunity to seasonal flu strains, however Covid-19 was a new virus to which no one to date has immunity (Ahn et al., 2020; Di Gennaro et al., 2020; Lingeswaran et al., 2020; Malik et al., 2020). Since Covid-19 was identified, it has resulted in widespread global morbidity and mortality (Sette & Crotty, 2021).

Covid-19 presented in the clinical setting with a combination of symptoms such as a fever, a dry cough and tiredness (Guan et al., 2020; Huang et al., 2020; Lingeswaran et al., 2020; World Health Organisation, 2020a). Recent studies show that a majority of the Covid-19 cases that result in mortality have underlying comorbidities (Richardson et al., 2020; Djaharuddin et al., 2021). Furthermore, it was found that the elderly (aged 70 years and older) and persons (any age group) who have serious underlying health conditions (e.g. hypertension, diabetes, cardiovascular disease, chronic respiratory disease and cancer) might be at higher risk for severe illness from Covid-19 (Lingeswaran et al., 2020; Lithander et al., 2020; World Health Organisation, 2020b).

The World Health Organization (WHO) issued stringent protective measures to be implemented in every country, calibrated to their capacity and context, to curb transmission and reduce the death rate associated with Covid-19. The measures included adopting behaviours

such as washing hands, avoiding touching their face, practicing good respiratory etiquette, individual level distancing, isolating in a community facility or at home if they are sick, identifying themselves as a contact of a confirmed case when appropriate, and cooperating with physical distancing measures and movement restrictions when called on to do so, (World Health Organisation, 2020b)

South Africa reported the first Covid-19 case on the 5th of March 2020 and as the cases increased, it was traced to predominantly South Africans who had travelled mainly from Europe (National Department of Health, 2020a). The South African government instituted a nation-wide, stay-at-home, lockdown for 21 days from 26 March in an attempt to curb community spread of the disease (National Department of Health, 2020b). This disruption to normal day-to-day lives meant that trade and commerce that were not deemed essential services were brought to a halt with immediate effect (The Presidency of South Africa, 2020). The South African government implemented some of the standard public health measures that were comprised of non-pharmaceutical interventions such as quarantine, social distancing and self-isolation (National Department of Health, 2020a, 2020b). Non-pharmaceutical interventions refer to a group of measures that individuals or whole communities can implement to curb the spread of the disease. These measures are often recognised as the first, most readily available line of defense against pandemic outbreaks (World Health Organisation, 2019).

With the onset of the Covid-19 pandemic and the 21-day stay at home lockdown, concerns unique to South Africa emerge that might hinder curbing community spread of the disease. Overcrowding in townships, in informal settlements and in shopping malls (queuing for food or social grant money) are major concerns in the prevention of the community spread of Covid-19 in South Africa (Department for International Development, 2020; News24, 2020). People from overcrowded townships and informal settlements may already be immunocompromised as their living conditions and environments place them at greater risk of infection (Bulled & Singer, 2020). Other already immunocompromised groups may include sex workers, drug users, homeless people and people with mental health disorders who struggle to access healthcare support. These even harder to reach groups reduce the effectiveness of public health interventions and processes aimed at containment. Moreover, anticipated stigma may create a barrier to access healthcare provision for a wide range of marginalised and already vulnerable groups (Department for International Development, 2020).

For marginalised and already vulnerable communities structural barriers often impede access to essential services such as healthcare (Logie & Turan, 2020). With the outbreak of Covid-19, resources have largely been diverted towards tackling the pandemic. For people with disabilities, there is an expectation that access to basic services including access to healthcare services and information during the Covid-19 outbreak go unhindered (World Health Organisation, 2020b). However, at the same time achieving this in practical terms will be difficult as some people with disabilities rely on others for their day-to-day needs and care (The Office of the United Nations High Commissioner for Human Rights, 2020). In addition, with the onset of the Covid-19 pandemic in South Africa, there has been much concern and speculation about the catastrophe that awaits once Covid-19 establishes itself in the poorest communities of South Africa and importantly, in the informal settlements (Boffa et al., 2020). Moreover, South Africa has the highest number of people living with HIV globally who are currently on anti-retroviral treatment (ART) (Simbayi, et al., 2018). According to Harries et al. (2020), how people living with HIV on ART will fare when infected by Covid-19 is unknown. In this paper, key informants from various sectors were recruited to explore how the Covid-19 pandemic has affected the vulnerable and marginalised groups. This approach was taken in lieu of concerns raised from various sectors where challenges were being experienced. Furthermore, this approach was used to potentially inform public health entities about the experiences from these particularly vulnerable groups for planning and prevention measures implemented with regard to Covid-19.

## Materials and methods

### Study setting and sample

Data collection took place between the 5th andthe18th of April 2020 in three provinces, namely Gauteng (GP), KwaZulu-Natal (KZN) and the Western Cape (WC) as these provinces had the highest burden of the disease at the time. In total, 60 key informants were purposively identified and selected as key informants from various South African sectors which assisted in assuring diversity of key informants representing various sectors of society. One focus group discussion was conducted. With regards to the gender breakdown: 36 cisgender women, 22 cisgender men and two transgender women took part in key informant interviews. The focus group discussion comprised of two cisgender women and three cisgender men. A summary of the key informant’s profile is provided in Table 1.

**Table 1.** Key informants profile.

KIIs	WC	GP	KZN	KIIs	WC	GP	KZN
Taxi owners/drivers/commuters	2	2	2	Sexual and gender minorities	2	2	0
Faith-based leader	1	1	1	NGOs for the homeless	2	0	0
Traditional leader	0	1	1	Migrant communities	1	2	2
Educators	1	1	1	People with disabilities	2	2	2
*Shebeen* (i.e. informal drinking place) patrons	2	2	1	Sex workers	1	1	0
People living with HIV	1	1	1	Out of school youth	2	2	2
Person living with chronic diabetes	1	0	0	Airport workers	1	1	1
Pregnant woman	1	0	0	Community health workers	2	2	2
Old age carers	1	1	4^a^				

^a^ A focus group discussion with four old age home carers.

Key informants were selected through existing collaborations with non-governmental organisations servicing particularly vulnerable and marginalised groups highlighted in Table 1.

### Data collection methods and procedure

Due to the highly infectious nature of Covid-19, all interviews were conducted telephonically. All interviewees received the study information sheet and consent form electronically either via email or WhatsApp message. Prior to the start of the interview, the researcher read out the information sheet and consent form to study participants. All questions posed by study participants were answered and verbal consent was obtained from study participants. On average, each interview lasted approximately 30–45 minutes. All interviews were audio recorded. Each researcher utilised their personal cellular telephones to conduct the interviews in a secure environment and their laptops to audio record the interviews. Interviews were conducted in a language of the key informant’s choice including English, isiXhosa, isiZulu and Afrikaans. All study participants received a reimbursement of ZAR 30 (USD1.63) airtime for their cellular telephone as a token of appreciation. Once completed, researchers transcribed the interviews verbatim and translated them if needed. All translations were back translated to verify accuracy. Finally, the study co-investigators verified the transcription and/or translation.

### Themes and probes

The themes that emanated from the key informant interviews and the focus groups conducted were guided by the main aim of the study as well as the following objectives: (a) to explore the general knowledge, perceptions and attitudes as well as behaviours with regards to Covid-19 among South African community members; (b) To gather the people from South African communities perception’s and reactions to Covid-19; (c) To understand and explore the daily lives of South African community members especially around income as well as coping strategies as it relates to the Covid-19 pandemic. The core questions as well as sub-category questions posed to the participants are listed in Table 2.

**Table 2.** Core questions and sub-categories.

**General Questions**	Knowledge of coronavirus and sources of information
	Community member reaction to the outbreak
	Precautions that have been taken
	Concerns
	Myths and conceptions
**Prevention**	Importance of protective measures
	Adherence to protective measures by community members
	Government’s response
**Impact of coronavirus**	Employment
	Livelihood
	Coping and managing infections

### Data analysis and management

Semi-structured interview guides were used to conduct key informant interviews. The following were explored in key informant interviews: (a) basic knowledge regarding the spread of Covid-19; (b) sources of information about Covid-19; (c) myths, misconceptions, false information about the spread of Covid-19; (d) perception of risk and (e) challenges experienced during the stay-in-place lockdown. Data were de-identified during the translation and transcription process. Atlas.ti.8 Windows was used to facilitate data analysis. The methodology underpinning this study was thematic analysis. Data analysis consisted of open coding using the participants’ own words, examining language used by study participants, categorising the information and using theoretical coding. In the first step of analysis, three members of the research team independently assigned codes to the transcripts. The team would then systematically group similar codes and independently place them into categories. The second step involved a discussion and consensus on the final categories to be kept for analysis. The themes were then drawn out from a process of comparative analysis of the primary codes against the categories identified and agreed to in the second step. The lead author and second co-author together with the principal investigator of the study led the data analysis process. Since open coding was used to identify the themes, it allowed this study to use participants own words and phrases without the interference of preconceived classifications. Standardising the coding by applying labels such that the lead authors could identify topics/themes/ideas and then carefully generating analytical schema allowed for comparison of the categories and facilitated interpretation of the findings.

### Reflexivity

Whether the researcher is an insider, sharing the characteristic, role or experience under study with the participants, or an outsider to the commonality shared by participants, the personhood of the researcher, including her or his membership status in relation to those participating in the research, is an essential and ever-present aspect of the investigation, according to Dwyer and Buckle (2009). All researchers who conducted interviews were proficient in each of the major languages spoken in each of the provinces where the study took place. Overall, researchers were able to reflect on some similarities experienced by study participants because of commonalities based on language, race and culture making them an ‘insider’ to some extent, however, all researchers completed a post-graduate education, which placed them as middle class, reinforcing an ‘outsider’ status, to many of our participants’ working-class situation (Schmidt et al., 2020). Fourteen researchers employed at the HSRC, all trained in qualitative data collection methodologies conducted the interviews. Researchers were proficient in each of the major languages spoken in the provinces where the study took place. In each of the participating provinces, 9 cisgender women and 5 cisgender men conducted key informant interviews, with one of the 14 researchers facilitating the focus group discussion (Schmidt et al., 2020). Of the 14 researchers, 4 completed doctorates in public health, anthropology, sociology and research psychology. The remaining 10 were all Masters-level educated researchers (Schmidt et al., 2020).

### Theoretical framework

The phenomenological approach in a general non-philosophical sense is grounded in qualitative methodology. Phenomenology is the study of the structure, and the variations of structure of the consciousness to which anything, event or person appears. Phenomenology focuses on clarifying both that which appears and the manner in which it appears. Phenomenology attempts to get beyond immediately experienced meanings in order to articulate the pre-reflective level of lived meanings to make the invisible visible (Turner, 2003). The phenomenological approach is used to gain a deeper understanding of the nature or meaning of the everyday ‘lived’ experiences of people and for the specific purpose of the study was used as the theoretical framework underpinning the methodology used as it provided the researchers the opportunity to probe the experiences with regard to the Covid pandemic and how it affected the interviewee’s lives.

### Ethical considerations

Ethics approval was obtained from the Human Sciences Research Council (HSRC) Research Ethics Committee (REC) (Protocol No REC 5/03/20). Due to the infectious nature of Covid-19, the ethical approval was granted subject to both participants and researchers being protected from the virus. To comply with the REC, all key informant interviews were conducted telephonically. Verbal consent was obtained for the interview as well as for the audio recording. To ensure confidentiality and the privacy of participants, only pseudonyms were used during data collection.

## Results

Three themes emerged from the data analysis and include the following: (1) knowledge, awareness and concerns for who is most vulnerable to Covid-19 in South Africa; (2) living in unstable environments with Covid-19 and (3) side lining of healthcare services during the Covid-19 pandemic.

### Knowledge, awareness and concerns for who is most vulnerable to Covid-19 in South Africa

I think that is because people think that certain people are immune to the virus and certain age groups are also immune to the virus. But then as the Coronavirus progresses we got to learn that even kids as young as 5 years old will be infected. It was no longer that thing of saying that it’s only older people that were being infected because I think people think only your certain age groups are at the risk of being infected and then they took it like it's far from us it will never get to us. But now the truth of the matter is that anyone who is living who is alive can get infected. (Pregnant woman: Gauteng)

How people construct knowledge and awareness of who is most vulnerable to Covid-19 in South Africa was informed by who was first infected by the virus. As is evident from the above quotation it appeared that key informants perceived ‘certain age groups [to be] immune to the virus’. This was because older people and international travellers were mainly infected with the virus. However, as Covid-19 spread, it seemed that notions of ‘certain age groups being immune to the virus’, slowly disappeared, and that there is an overall understanding that everyone is at risk of becoming infected. Data suggest that key informants had a high level of knowledge and awareness of who is most vulnerable to Covid-19. Across key informant interviews, study participants reported that they are most concerned for people who are over the age of 70 becoming infected, since they are more vulnerable to Covid-19:

It would be my mother-in-law because she is in her 80s. She is in an old-age unit where she is, they are on lockdown. They cannot even come up and have their breakfast and lunch. So, she is the one who I am mostly concerned about. (Person with a disability: Gauteng)

 … I am in the age group of 70. I will speak now for my community and by [in] large, the community where I live. [We are] tak[ing] the virus very seriously and the [possible] effects of the virus in our community. We have quite a few old age institutions that are housing older people that are in my group and I think the community as a whole took the virus very seriously. (Person with chronic illness: KwaZulu-Natal)

People with pre-existing health conditions (including chronic illness) according to key informants are more at risk of being infected with Covid-19:

So those are the people that I am concerned about and the elderly in the main. We are a country that is the most unequal one and we have the highest number of people living with communicable diseases. So most of us are immunocompromised those who are suffering from HIV/AIDS and TB. (Traditional leader: Gauteng)

It is those who are HIV positive, also pulmonary TB that person is at high risk, people with high blood [pressure], asthmatic people are also at risk. (Community health worker: Western Cape)

Due to the identification of certain groups of people as more vulnerable to Covid-19 as elaborated by the participants in the statement above, people with pre-existing health conditions and disabilities and those who provide care services for the elderly are in a constant state of fear of infection:

Yes, it is very important for me because I am living with a disability. I have a [weak] respiratory system. I am very much vulnerable so … I have to take 10 times the measure. (Person with a disability: Gauteng)

Especially when the state president announced the lockdown. That was it for me. I [had] [an] anxiety attack because of my chronic illness.’ – Person living with HIV: KwaZulu-Natal

*‘*The fear will kill me before this virus. (Old age home carer: Western Cape)

Stringent precautionary measures to protect the elderly from infection were followed at old age homes. This was expressed in a focus group discussion at an old age home in the following manner:

We were proactive. We started the process long before the lockdown. Especially, we cut down on visitation rights to nil and we have reengineered the screening process. We learnt on an almost daily basis where initially we were just sanitizing and using masks and then we introduced the [laser thermometer] and that was done at the security hut. Now we have gone a little further, at the clock-in points, we also check temperatures and sanitize again. So, as we [got] more educated, we filter[ed] this education to our staff. (Old age home carers: KwaZulu-Natal)

South Africa has the highest number of people living with HIV globally. In our study, key informants echoed the concerns of public health specialists of the effect of Covid-19 on people with suppressed immune systems. People living with HIV shared similar fears of becoming infected and potentially dying from Covid-19:

The way that I understand it people who are living with HIV and TB that [the virus] is very dangerous for them. So people [who] live with these illnesses that [the virus] can also [be the] cause [of] your death and so as [for] myself who is HIV-positive. I am not saying that I am afraid to die but I am afraid because this is a deadly virus and look I am HIV positive. We have a big chance and we have many years to live … but I feel that if we get into contact with this virus then it will [be the] cause of [my] death. While antiretroviral treatment gives us a life. Before the lockdown we decided that we will protect ourselves from the outside world and from communities. (Person living with HIV: Western Cape)

### Living in unstable environments with Covid-19

Social and physical distancing are proven measures to curb the spread of Covid-19. People living in overcrowded townships and informal settlements may already be immunocompromised as their living conditions and environments place them at greater risk of infection. These were also concerns raised by study participants that social and physical distancing might be challenging for those living in townships and in overcrowded informal settlements:

Most likely is the poor people because the middle and the more established people, we can self-quarantine at home. But the poor people do not have money for soap and food. It’s very hard and its very bad in the townships and locations they will be more infected because there is no proper social distancing. How do you expect people to do that if they liv[e] in communal shacks and stuff like that? (Airport worker: Gauteng)

People who [live] in shacks, in townhips, because they stay in high number[s] in [a] single shack and they still go around for food and do business as usual. (Taxi commuter: Western Cape)

Access to water and or the use of communal taps as well as the lack of access to sanitisers were also concerns raised by key informants that puts already immunocompromised groups at risk:

No. I don’t think the government is doing enough at all. Firstly, because there are places in Philippi that only uses one tap for the community. So can they expect people to wash with that type of situation? How do [they] expect people to maintain hygiene when they have one tap? (Shebeen patron: Western Cape)

What worries me the most about the coronavirus is that people in the rural areas do not have access to sanitizers and gloves that people are expected to use. We also need mobile clinics to do the testing. (Traditional leader: KwaZulu-Natal)

### Sidelining of healthcare services

Of particular concern for people over the age of 70 and people with comorbidities were concerns of stock-outs and being able to access healthcare during the lockdown. The fears of stock-outs and side lining of healthcare services emerged as an added fear for vulnerable, immunocompromised groups:

I contacted one of the private hospitals for medication and they told me it will only be given to in-patients who have prescriptions. So unfortunately, mine is over the counter and I cannot get some of the medications. So, getting [my medication] is a problem. Sometimes they are out, and these are essential for me and it is a problem. (Person living disabilities: Gauteng)

We understand the president said only emergencies at trauma units, what about our communities or needs … everything now [has] been put to the side. (Person living with HIV: Western Cape)

Not having the means to access medication was mentioned by key informants as a concern; however not being able to access healthcare services increased anxieties and added to feelings of hopelessness:

Okay, what I know about this thing is it’s affecting more people in South Africa especially, the people they got a problem already like us with our immune system that has been compromised. Already my worry is that how are we going to survive if we do not get help from our clinics. How are we going to survive if there is nobody from the health department who is walking around showing us what to do, because what we are doing in our houses, I am sure is not enough? We don’t even know where to go and what to do, when you go to the clinic. (Person living with HIV: Gauteng)

Yes, obviously like on a lockdown day [the homeless people] who were [at the shelter] [were kept there]. We tried to get new people [in] but they won’t take any, like we found all the services, sub services, in-patient programs for mentally ill, everybody locked their door and said we are not taking anyone. (Representative of an NGO providing services to homeless people: Western Cape)

As is evident from the above quotation already vulnerable groups such as homeless people and people with mental disabilities were further marginalised as operations and services were restricted or shutdown when the stay at home lockdown was implemented. Cisgender women may not be receiving the adequate support with regard to family planning services when they eventually make their way to clinics, especially during the lockdown:

They are actually turning away women [who] are coming [to the clinic] for family planning. Just like they tell us that, they are not doing this during this time and they give you a pack of condoms without even asking if your partner uses these condoms or you prefer female condoms [or] whatever. I am talking about what was happening last week right now. (Person living with HIV: KwaZulu-Natal)

Similarly, community health workers expressed a concern for not being able to do follow-up visits and counselling with their clients because of the movement restrictions, as is evident from the below quotation:

As I said before [the lockdown] has affected my work, because I work with sick people [who]needs visits for health issues and counselling. Now we cannot see them, we do not know if maybe one of them has [been infected with the] corona[virus]. Maybe one has given birth to a underweight child. We do not know such information because we cannot do our [home] visits because of lockdown. (Community health worker: Western Cape)

## Discussion

To date, South Africa had recorded 1538,451 confirmed cases of Covid-19 and 182,983 vaccines administered (23 March 2021) (National Department of Health, 2021). Non-pharmacologic approaches such as fluid support, oxygen and ventilatory support still remain a means of managing people affected (Fisher & Heymann, 2020). However, because of the roll-out of vaccines that have been set as a phased in process, increasing fears of infection remain ubiquitous. Without a vaccine available, the US Centers for Disease Control and Prevention (2020) reported that people with immunocompromised conditions might not be fully able to fight off Covid-19 and therefore be more susceptible to infection (Centers for Disease Control and Prevention, 2020). For elderly people, the risk of death was said to be high as their immune systems weaken with age, making it more difficult to fight off infections as chronic diseases were often found among the elderly as well (Lithander et al., 2020; Mash, 2020). In a rapid clinical review conducted by Lithander et al. (2020) elderly people with underlying diseases are more likely to be infected with the virus and develop severe disease.

Across key informant interviews, study participants reported that they are most concerned for people who are over the age of 70 since Covid-19 poses a high risk to older people. Moreover, we found that caregivers of elderly people also expressed concerns for fear of infection. In South Africa, with the onset of the pandemic, several residential facilities for elderly people have recorded Covid-19 infections and deaths (Kassen, 2020). For example in Cape Town, at Highlands House, in Vredehoek, 11 residents and 27 staff members were reported to have tested positive for Covid-19 (Evans, 2020). These Covid-19 infections and deaths at residential facilities for elderly people, raised anxiety and fears amongst the caregivers of older people. In our study, caregivers of elderly people, echoed fears of concern for elderly people becoming infected with Covid-19.

With the advent of the Covid-19 pandemic in South Africa, particular concerns were raised for people living with HIV. However, Phaswana-Mafuya et al. (2020) reported that the impact of Covid-19 on people living with HIV is not yet clear within South African contexts. Our study found that people living with HIV had increased fears of becoming infected with SARS-CoV-2, despite no known scientific evidence that people living with HIV are more vulnerable.

The South African government adopted measures issued by the WHO and set further regulations in place, which included an extended stay-at-home lockdown where only essential workers and services were allowed to operate (The Presidency of South Africa, 2020). The extended lockdown severely affected many small-owned business and daily wage earners, already struggling to feed and support their families, across the country (Centre for Development and Enterprise, 2020). The Covid-19 pandemic and the resultant lockdown and movement restrictions has not only deepened the socio-economic inequalities in South Africa but has further marginalised vulnerable and underserved communities (SANEWS, 2020). These socio-economic inequalities have become even more pronounced with the advent of the Covid-19 pandemic highlighting how impoverished conditions, such as overcrowding and poor sanitation contributes to the vulnerability of infection for people living in already unstable environments (Fisher et al., 2020). Our study findings also brought attention to this, in that key informants expressed concerns for family members and school-going children living in overcrowded townships with poor sanitisation and lack of access to water.

Access to healthcare services is important for vulnerable and immunocompromised people and the effect of quarantines, lockdowns and social distancing should not be a hindrance to accessing healthcare services including medication (AGE Platform Europe, 2020). The Office of the United Nations High Commissioner for Human Rights (2020) argued that countries must increase social protection measures in order to ensure that underserved and vulnerable groups are supported in a safe mode throughout the crises. This study found that the concern of essential health needs was not being met, namely access to medication (including chronic medication) – that was said to be limited; services at clinics that were no longer accessible and in some instances people being turned away from reproductive healthcare services. In a qualitative study conducted in Zimbabwe, healthcare workers expressed concerns that a singular focus on prevention and treatment of Covid-19 would lead to the critical needs of those suffering with other diseases being neglected (Mackworth-Young et al., 2020). Healthcare workers indicated that without access to prevention measures for other diseases, such as condoms, an increase in the incidence of STIs, unintended pregnancies and HIV was predicted (Mackworth-Young et al., 2020; Riley, Sully, Ahmed, & Biddlecom, 2020) Futhermore, lack of access to family planning on unintended pregnancies and the consequences thereof was raised as a particularly important issue (Mackworth-Young et al., 2020).

### Strengths and limitations

The results of this study should be considered in terms of its methodological limitations. Due to the qualitative research design, only very tentative generalisations of the study findings are possible. Qualitative research sheds light on the needs of specific marginalised groups during health crises and infectious or chronic illness epidemics (Teti, Schatz, & Liebenberg, 2020). Marginalised communities vulnerabilities to disease are not just biological but social, qualitative in-depth research has the potential to provide critical information (Teti, Schatz, & Liebenberg, 2020).

Telephonic interviews are usually seen as a versatile method in attaining responses from key individuals regarding their perceptions, opinions and attitudes about specific issues. However, in social science research when certain topics are of a sensitive nature, body language is often relied on when engaging in face-to-face interviews but not possible when conducting telephonic interviews. Therefore, the limitation experienced during this study was that interviewers had to rely solely on the responses provided by participants, and in some instances, these responses were short, with little elaboration, even with probing. Frustrations around connection and clarity were expected, however it remained a cumbersome issue to deal with during the interviews. Interviewers initially struggled identifying key informants because of the lockdown, as little to no pre-existing relationships were in place before the lockdown. Cold calls had to be relied on which ultimately influenced the gender balance of interviewees recruited. Despite these limitations, this study brought attention, for the first time, to how vulnerable, immunocompromised groups and caregivers of the elderly experience and perceive the onset of the Covid-19 pandemic in South Africa.

## Conclusion

Following the first reported case in March 2020, SARS-CoV-2 cases increased to 196,750 confirmed cases and 3199 deaths as of 13th July 2020 (National Department of Health, 2020b). Synonymous with day-to-day updates on the number of cases and deaths reported is the continual communication about who is most vulnerable to Covid-19 globally and in South Africa. In this way, fear of infection becomes ubiquitous for older people and people with underlying health conditions with the advent of the Covid-19 pandemic. In South Africa, our data demonstrated that people living with HIV had an increased fear of infection to Covid-19 despite no evidence suggesting that they are more vulnerable.

Fears of infection and anxiety brought on by the Covid-19 pandemic impacts on the mental and emotional health of vulnerable and immunocompromised groups such as people living with HIV, the elderly and people with comorbidities. Therefore, it becomes particularly important for public health professionals to engage vulnerable, immunocompromised groups in decision-making processes for response, recovery, preparedness and risk reduction. In this way, government and policymakers will have an in-depth understanding of how the Covid-19 pandemic affects vulnerable and immunocompromised groups and their caregivers, potentially resulting in improved systemic institutional involvement in structural changes that will benefit these vulnerable groups.
